# Structure and Mechanism of PhdC, a Prenylated‐Flavin Maturase

**DOI:** 10.1002/prot.70096

**Published:** 2025-12-09

**Authors:** Dominic R. Whittall, Henry G. Box, Karl A. P. Payne, Stephen A. Marshall, David Leys

**Affiliations:** ^1^ Manchester Institute of Biotechnology, University of Manchester Manchester UK

**Keywords:** cofactor maturation, enzyme mechanism, enzyme structure, flavin, oxidation, prFMN

## Abstract

Prenylated flavin mononucleotide (prFMN) is a modified flavin cofactor required by the UbiD family of (de)carboxylase enzymes. While the reduced prFMNH_2_ form is produced by the flavin prenyltransferase UbiX, the corresponding two‐electron oxidized prFMN^iminium^ form is required to support UbiD catalysis. Thus, oxidative maturation of prFMNH_2_ is required, which can be catalyzed by UbiD. However, heterologous (over)expression of UbiDs frequently leads to the accumulation of the stable but non‐active one‐electron oxidized purple prFMN^radical^ species. A dedicated prFMN maturase enzyme (PhdC) from *Mycolicibacterium fortuitum* was recently identified as capable of catalyzing the oxidative maturation of prFMN^radical^ to prFMN^iminium^, thereby enabling an effective supply of active cofactor to the associated phenazine‐1‐carboxylate (de)carboxylase PhdA. We report the crystal structure of PhdC in complex with flavin, revealing it is a distant member of the class I HpaC‐like family of short‐chain dimeric flavin reductases and demonstrate catalytic conversion of the prFMN^radical^ species to prFMN^iminium^ in the presence of oxygen or ferricyanide. Co‐expression of PhdC or a distant homologue from *Priestia megaterium* (YclD) with the canonical UbiD from *Escherichia coli* leads to activation of the latter, similar in effect to co‐expression with the prFMNH_2_‐binding chaperone LpdD. Conserved Glu residues in the PhdC active site suggest catalysis occurs through C1′ proton‐abstraction coupled oxidation. This study thus provides both structural and mechanistic insight into the function of PhdC, adding to the expanding repertoire of prFMN‐binding proteins associated with the widespread UbiDX system.

**Abbreviations**
BLAST: basic local alignment search tool
CCP4: Collective Computational Project no. 4
ClusteredNR: clustered non‐redundant
DMAP: dimethylallyl phosphate
DMSO: dimethyl sulphoxide
DSF: differential scanning fluorimetry
EDTA: ethylenediaminetetraacetic acid
Fe‐CN: ferricyanide
FMN: flavin mononucleotide
HPLC: high‐performance liquid chromatography
IPTG: isopropyl β‐D‐1‐thiogalactopyranoside
ITC: isothermal titration calorimetry
MAFFT: multiple alignment using fast fourier transform
NADH: reduced nicotinamide adenine dinucleotide
Ni‐NTA: nickel‐nitrilotriacetic acid
PEG: polyethylene glycol
prFMN: prenylated FMN
rpm: revolutions per minute
TFA: trifluoroacetic acid
UV: ultraviolet

## 1 Introduction

**FIGURE 1:**
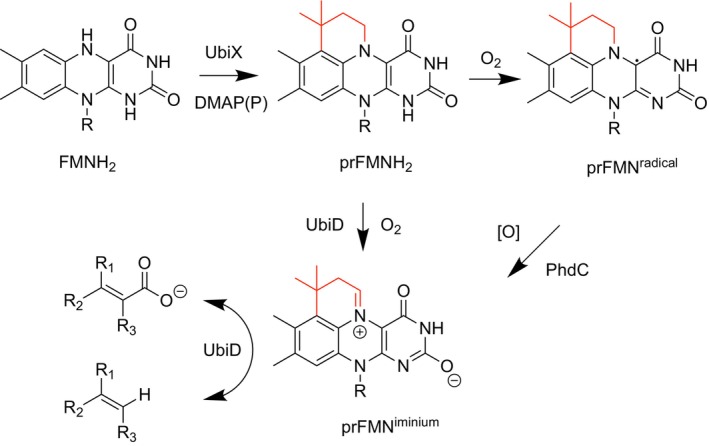
prFMN synthesis and oxidative maturation. Schematic representation of the UbiX‐mediated biosynthesis of reduced prenylated FMN (prFMNH_2_) and ensuing two‐electron oxidative maturation to the active prFMN^iminium^ cofactor within the reversible (de)carboxylase UbiD [3]. In contrast, one‐electron oxidation to the inactive prFMN^radical^ state occurs when free in solution or bound to UbiX or certain UbiD‐family members [4]. PhdC‐mediated oxidative maturation of prFMN^radical^ is proposed to provide a route to revert to the catalytically active prFMN^iminium^ species [5].

The UbiD family of enzymes is ubiquitous in microbes and catalyzes the reversible (de)carboxylation of a wide variety of (hetero)aromatic and unsaturated aliphatic substrates [1]. Catalysis is dependent on a modified form of flavin mononucleotide (FMN) through prenylation forming a 4th non‐aromatic ring [2]. Synthesis of this prenylated‐FMN (prFMN) is carried out by the associated UbiX family of flavin prenyl‐transferases which utilize dimethylallyl‐(pyro)phosphate and reduced FMNH_2_ to produce prFMNH_2_ (Figure 1) [6]. In the model UbiD enzyme Fdc1 from *A. niger* , prFMNH_2_ must bind to the active site prior to exposure to molecular oxygen in order to undergo correct oxidative maturation to the active prFMN^iminium^ species [7]. Formation of the prFMN^iminium^ is essential for supporting the 1,3‐dipolar cycloaddition mechanism during the Fdc1 (de)carboxylation cycle [3]. Although alternative mechanisms have been proposed for other UbiD enzymes, all are postulated to require prFMN^iminium^ [8]. However, if prFMNH_2_ is exposed to O_2_ prior to binding to Fdc1 (either in solution or still bound to the prenyltransferase UbiX), it readily forms a stable purple radical (prFMN^radical^) species, which Fdc1 is unable to mature into prFMN^iminium^ [9]. While Fdc1 is capable of efficient autocatalytic maturation both in vivo and during reconstitution in vitro, other heterologously expressed UbiD enzymes appear to be less efficient in this process, either accumulating a mixture of oxidized prFMN species or, in the case of the canonical *Escherichia coli* UbiD, exclusively yielding the prFMN^radical^ complex [4].

A prFMNH_2_‐binding chaperone LpdD, associated with the gallic acid (de)carboxylase LpdC, has been shown to support limited activation of *E. coli* UbiD protocatechuic acid decarboxylation activity through co‐expression. However, no evidence was found that LpdD has a direct catalytic role in cofactor maturation [10].

More recently, studies of the phenazine‐1‐carboxylate decarboxylase from *Mycolicibacterium fortuitum* have shown that it is expressed as part of a three‐gene operon consisting of the (de)carboxylase (*phd*A), the flavin prenyltransferase (*phd*B), and a gene of unknown function (*phd*C) [5, 11]. Co‐expression with *phd*C and *phd*B was shown to significantly increase the activity of recombinant PhdA compared with the co‐expression of *phd*B alone [5, 12]. It was subsequently demonstrated that purified PhdC was capable of oxidizing the prFMN^radical^ to the catalytically active iminium form in vitro (Figure 1). Additionally, PhdC facilitated prFMN^iminium^ incorporation in heterologously expressed 2,5‐furandicarboxylate (de)carboxylase HmfF. Taken together, this activity suggests PhdC is capable of functioning as a dedicated prFMN maturase [5]. Herein, we report the crystal structures of PhdC in the *apo*‐ and FMN‐bound states. Together with supporting solution data, these structures enable us to propose a mechanism for PhdC‐mediated oxidation of the inactive prFMN^radical^ to the catalytically active species, prFMN^iminium^.

## 2 Materials and Methods

### 2.1 Cloning of PhdC for *E. coli* Heterologous Expression

The *Mycolicibacterium fortuitum phdC* gene was codon optimized for expression in *E. coli* and synthesized as a gene‐string (GeneArt). The gene‐string was designed with 15 bp 5′ and 3′ extensions that were complementary to the *Nde*I and *Xho*I sites of pET30a (Merck) to allow direct cloning of the gene‐string into linearized pET30a using In‐Fusion HD (Clontech) to produce MfPhdCpET30a, an expression plasmid for production of C‐terminally His‐tagged protein. Once the sequence of the insert was verified, the purified plasmid was transformed into the *E. coli* BL21(DE3) expression strain (New England Biolabs). For co‐expression of His‐tagged PhdC with UbiX, MfPhdCpET30a was co‐transformed with a plasmid encoding untagged *E. coli ubiX* in pET21b as described previously [3].

To allow co‐expression of UbiD (de)carboxylases with both untagged *phdC* and *ubiX*, the latter two genes were cloned into pBbA1c to form an operon [13]. First, *E. coli ubiX* was amplified with pBbA1cEcXF (CGGTGCTTAAGGATCCACAAGGAGGAATCGACATGAAACGACTCATTGTAGGC) and pBbA1cEcXR (GAGATCCTTACTCGAGTTATGCGCCCTGCCAGCG), the former encoding an AGGAGG ribosome binding site (underlined) upstream from the *ubiX* start codon. The resulting PCR product was cloned into pBbA1c linearized at the *Bam*HI/*Xho*I sites using In‐Fusion HD(Clontech) to generate RFPEcUbiXpBbA1c containing a two‐gene operon with the plasmid‐encoded RFP gene in the first position followed by the EcUbiX encoding gene. Once sequence verified, RFPEcUbiXpBbA1c was linearized at the *Nde*I/*Bam*HI sites to remove the *rfp* gene and allow *phd*C amplified using primers PhdC_EcXA1F (gaaggagatatacatatgATGATGAGCAAAGTTCCGGGTG) and PhdC_EcXA1R (tcctccttgtggatcc **TTA**CTGCTGTTCATAACCTGCACGC) to be cloned in its place to produce plasmid MfPhdCEcUbiXpBbA1c. A PhdC homologue, YclD, was identified in *Priestia megaterium* DSM319 (WP_013083431). The gene was PCR amplified from *P. megaterium* genomic DNA using primers YclD_EcXA1F (GaaggagatatacatatgAATAAAGCAGAGCTGCCAAAAGCC) and YclD_EcXA1R(tcctccttgtggatccTTAAGAATCGGTTTTTTCAGTAGAAAGAACAGC) and cloned into linearized RFPEcUbiXpBbA1c as above to produce *Pm*YclD EcUbiXpBbA1c. For co‐expression with (de)carboxylases, the above plasmids were co‐transformed into BL21(DE3) with either EcUbiDpET28a (for *E. coli* UbiD^8^ [4]) or AnFdc1pET30a (for *Aspergillus niger* Fdc1 [3]), with successful transformants selected for using 50 μg/mL kanamycin and 34 μg/mL chloramphenicol.

### 2.2 Heterologous Expression of *M. fortuitum* PhdC

*E. coli* transformants were grown in terrific broth supplemented with 50 μg/mL kanamycin at 37°C/180 rpm until the culture reached an OD_600_ ~0.6–0.8. Cultures were induced with 0.25 mM isopropyl ß‐D‐1‐thiogalactopyranoside (IPTG) and grown overnight at 17°C/180 rpm. Culture were harvested by centrifugation (4°C, 7000 *g* for 10 min) and pellets stored at −20°C.

### 2.3 Purification of *M. fortuitum* PhdC

Cell pellets were resuspended in buffer A (200 mM NaCl, 50 mM Tris/Cl pH 7.5) supplemented with RNAse, DNase, and SigmaFast ethylenediaminetetraacetic acid (EDTA)‐free protease inhibitor cocktail (Sigma). Cells were lysed using a continuous cell disruptor (Constant Systems) at 20 000 psi, and the lysate was clarified by ultracentrifugation at 125 000 *g* for 60 min. The supernatant was applied to a gravity flow column containing 5 mL of nickel‐nitrilotriacetic acid (Ni‐NTA) agarose resin (Qiagen). The resin was washed successively with 3 column volumes of buffer A supplemented with 10 and 40 mM imidazole, respectively, and protein was eluted in 1 mL fractions using buffer A supplemented with 250 mM imidazole. Samples were subjected to SDS‐PAGE analysis, and fractions found to contain the purified protein were pooled. Imidazole was removed using a 10‐DG desalting column (Bio‐Rad) equilibrated with buffer B (100 mM NaCl, 25 mM Tris pH 7.5).

### 2.4 UV–Vis Spectroscopy/Protein Quantification

UV–Vis absorbance spectra were recorded with a Cary 50 Bio UV–Vis spectrophotometer using assay buffer (100 mM KPi, pH 7.5). Protein concentration was estimated using ε_280_ = 8605 M^−1^ cm^−1^ (calculated from the primary amino acid sequence using the ProtParam program on the ExPASy proteomics server) [14]. Samples containing PhdC bound to FMN were prepared by incubating *apo*‐PhdC with excess FMN for 60 min at room temperature prior to the removal of unbound FMN via desalting into assay buffer.

### 2.5 Production of prFMN

The prFMNH_2_ synthesis reaction was performed in 50 mM Tris buffer (pH 7.5) containing 200 mM KCl, 1 mM FMN, 2 mM dimethylallyl phosphate (DMAP; prenyl donor), 5 mM reduced nicotinamide adenine dinucleotide (NADH), 10 μM Fre reductase, and 50 μM UbiX (from *Pseudomonas aeruginosa* , heterologously expressed in *E. coli* as previously described [15]). The reaction was incubated overnight at room temperature under anaerobic conditions (100% N₂ atmosphere, Belle Technology glove box). After incubation, the mixture was passed through a 10 kDa molecular weight cutoff spin concentrator (Vivaspin) to remove UbiX and Fre proteins. The resulting prFMNH₂‐containing filtrate was analyzed using a UV–Vis spectrophotometer housed in the anaerobic glove box. Reconstitution of PhdC with prFMNH_2_ was performed as previously described [15]. The oxidized species, prFMN^radical^, was formed by removing aliquots of prFMNH_2_ from the glove box and exposing them to air, or by diluting with oxygenated buffer until the reaction mixture turned purple. Samples were returned to the glove box and analyzed via UV–visible spectroscopy. Alternatively, prFMN^radical^ could be formed under anaerobic conditions in a glove box via addition of two molar equivalents of potassium ferricyanide (K_3_[Fe(CN)_6_]).

### 2.6 Enzyme Kinetics

Initial rates of prFMN^radical^ substrate consumption by PhdC were determined by monitoring the linear decrease in absorbance at 515 nm using a Cary 50 Bio spectrophotometer. Assays were performed within an anaerobic glove box (Belle Technology) against various apparent concentrations of prFMN^radical^ substrate in assay buffer at room temperature. The rate of PhdC‐driven oxidation of prFMN^radical^ to prFMN^iminium^ was determined using A_515 nm_ and the extinction coefficient of prFMN^radical^ (ε515 nm = 3.017 mM^−1^ cm^−1^, Figure S2). All rate values are apparent as the exact prFMNH_2_ content of the initial synthesis reaction cannot be accurately determined [7].

### 2.7 PhdC Activity With Alternative Oxidizing Agents

To ensure the complete absence of oxygen, assays were performed in the presence of glucose oxidase and catalase under a nitrogen atmosphere within the micro‐aerobic glove box. Reactions were performed using assay buffer supplemented with 10 mM glucose. PhdC and prFMN species were prepared as described elsewhere in the methods. Assays contained 20 U/mL glucose oxidase, 60 U/mL catalase (both from Sigma‐Aldrich), 100 μM prFMN species, 50 μM PhdC, and either 2 M equivalents of potassium ferricyanide (FeCN) or 1 mM nicotinamide adenine dinucleotide (NAD+), respectively. Species of prFMN were incubated for 30 min with PhdC prior to the addition of the oxidizing agent. Reactions were incubated for a further 30 min following the addition of the oxidizing agent.

### 2.8 Thermostability Assays

Thermostability measurements of PhdC in *apo*‐ and FMN‐bound forms were performed in assay buffer using a SUPR‐ differential scanning fluorimetry (DSF) instrument (Applied Photophysics). Samples were subjected to a linear temperature ramp of 1°C/min, with excitation at 280 nm and emission recorded from 310 to 390 nm. Melting temperatures (T_m_) were determined by fitting the fluorescence data using the instrument's packaged software.

### 2.9 Isothermal Titration Calorimetry

ITC was used to determine the binding affinity of FMN to PhdC using a MicroCal Auto‐iTC200 instrument (GE Healthcare). All experiments were carried out at 25°C in degassed assay buffer. PhdC was prepared at a concentration of 236 μM, and FMN was loaded into the syringe at a concentration of 2360 μM. Both protein and ligand solutions were buffer exchanged or prepared directly in the same degassed assay buffer to ensure consistency. The titration consisted of 15 sequential injections of 2.5 μL FMN into the PhdC solution, resulting in a final FMN concentration of 217 μM in the cell. Data were analyzed using the instrument's integrated software to determine the binding constant (*K*_*d*_).

### 2.10 Multiple Sequence Alignment and Phylogenetic Analysis

Homologues of PhdC were identified using the NCBI Basic Local Alignment Search Tool (BLAST) against the clustered non‐redundant (ClusteredNR) database [16]. The resulting alignment file was downloaded and visualized in Jalview for manual inspection [17]. Sequences exhibiting significant truncations at either the N‐ or C‐terminus (approximately ≥ 20 residues) were removed to ensure alignment quality. The *Priestia megaterium* YclD sequence was then added to the curated dataset, and a consensus sequence was generated in Jalview. The final multiple sequence alignment was used to construct a phylogenetic tree via the EMBL‐EBI webserver using the multiple alignment using Fast Fourier Transform (MAFFT) multiple sequence alignment tool under default parameters [18].

### 2.11 Whole Cell Assays

All *Ec*UbiD whole‐cell assays used *E. coli* T7 express cells, while *An*Fdc experiments were carried out in BL21. For negative controls, empty UbiD backbone vector pET28a and RFPEcUbiXpBbA1c were used. All the constructs were expressed in Luria‐Bertani Broth media containing appropriate antibiotics and induced with 0.5 mM IPTG as previously described. Harvested cells were resuspended in 50 mM KPi (pH 6.5) containing 150 mM NaCl to final optical densities of 10 for cells overexpressing EcUbiD or 1 for cells overexpressing AnFdc1 at 600 nm. Cinnamic acid stocks were prepared in absolute dimethyl sulphoxide (DMSO), and protocatechuic acid stocks were prepared in absolute ethanol, then added to reactions at 2% (*v/v*). Standard reaction volumes were 500 μL, with a final substrate concentration of 5 mM. Reactions were run for 18 h at 35°C and 350 rpm within an Eppendorf ThermoMixer. Reactions were quenched by adding an equal volume of acetonitrile containing 0.1% trifluoroacetic acid (TFA), followed by centrifugation at 16 000×*g* to remove precipitated proteins and cell debris. Supernatants were analyzed using an Agilent 1290 Infinity Series high‐performance liquid chromatography (HPLC) system. Chromatographic separation of cinnamic acid and styrene was performed on a Kinetex C18 column (5 μm, 100 Å, 250 × 4.6 mm) using an isocratic mobile phase of 50:50 (*v/v*) acetonitrile:water at a flow rate of 1 mL/min, while separation of protocatechuic acid and catechol used an isocratic mobile phase of 40:60 (*v/v*) acetonitrile:water.

### 2.12 Crystallization

Purified PhdC in buffer B was diluted to a final concentration of 20 mg/mL. Initial screening by sitting drop was performed using a selection of commercially available screens (Molecular Dimensions), mixing 0.3 μL protein with 0.3 μL mother liquor, and led to crystals in a variety of conditions when incubated at 4°C. FMN‐bound structures were obtained via co‐crystallization.

### 2.13 Diffraction Data Collection and Structure Elucidation

Crystals were briefly soaked in mother liquor supplemented with 10% polyethylene glycol PEG200 and flash frozen in liquid nitrogen. Diffraction data were collected at Diamond beamlines and processed using the CCP4 Collective Computational Project no. 4 (CCP4 suites) [19]. The structure was solved by molecular replacement implemented in Phaser [20] using an alphafold model for PhdC. Automated model building and refinement were carried out on the molecular replacement solution using Phenix.autobuild. Iterative cycles of manual model building in Coot followed by refinement using Phenix.refine were used to complete the structures [19, 21]. For diffraction and refinement statistics see Table 1.

**TABLE 1:** X‐ray diffraction data collection and refinement statistics.

	PEG‐bound PhdC (PDB: 9SRJ)	FMN‐bound PhdC (PDB: 9SRG)
Data collection		
Wavelength, Å	0.9762	0.9762
Space group	I 2 3	I 2 2 2
Cell dimensions		
a, b, c (Å)	121.25 121.25 121.25	59.73 75.80 79.43
Monomers per asymmetric unit	2	1
Solvent content, %	39.81	50.27
*CC1/2*	1.0 (0.3)	1.0 (0.6)
*I*/σ*I*	26.2 (0.4)	7.0 (1.4)
Completeness (%)	100.0 (100.0)	89.3 (78.0)
Redundancy	37.0 (16.5)	12.5 (7.7)
Wilson B factor (Å^2^)	23.3	9.9
Refinement		
Resolution (Å)	49.50–1.42 (1.44–1.42)	54.83–1.58 (1.64–1.58)
No. reflections	55801 (2807)	27304 (1365)
*R*_work_/*R*_free_	0.181/0.204 (0.380/0.374)	0.217/0.230 (0.247/0.262)
No. non‐hydrogen atoms	2616	1414
Mean B factor (Å ^2^)	35.0	13.31
R.m.s. deviations		
Bond lengths (Å)	0.009	0.011
Bond angles (º)	1.32	1.49
Ramachandran plot		
Favourable regions (%)	99.66	98.65
Allowed regions (%)	0.34	1.35
Outliers (%)	0	0

## 3 Results

### 3.1 Heterologous Production and Solution Characterization *M. fortuitum* PhdC

**FIGURE 2:**
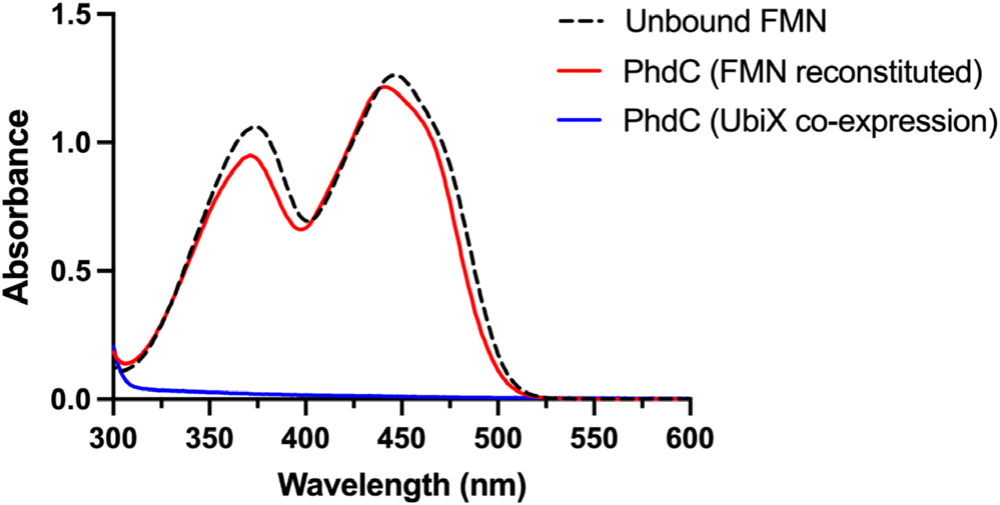
Spectral characterization of the PhdC‐FMN complex. UV–visible absorption spectra of PhdC co‐expressed with UbiX post‐purification (*apo* form, in blue) and after reconstitution with FMN (in red). Comparison with a spectrum of unbound FMN free in solution (in black) confirms a spectral shift occurs upon PhdC:FMN complex formation.

The *phdC* gene was cloned with a C‐terminal His tag, overexpressed in *E. coli* , and aerobically purified to homogeneity using Ni‐NTA affinity chromatography (Figure S1). Purified PhdC protein co‐expressed with UbiX possessed no visible color, and the UV–vis spectrum lacked any features normally associated with flavin or prFMN binding (Figure 2). We initially studied FMN‐binding as a proxy for prFMN‐binding, given the challenges associated with producing large amounts of pure prFMN species, many of which are unstable [9]. Following incubation of free FMN with purified PhdC and subsequent desalting to remove non‐specifically bound FMN, the corresponding spectrum underwent a slight blue‐shift toward ~440 nm, suggestive of PhdC‐FMN complex formation (Figure 2). Unlike UbiD, but akin to UbiX [4, 22], PhdC does not appear to require Mn^2+^ for flavin binding. The PhdC binding affinity (*K*_*d*_) for FMN was determined using isothermal titration calorimetry (Figure S3, Table S1). FMN displayed a *K*_*d*_ of 17.75 ± 0.26 μM, comparable to/consistent with values reported for other flavin‐binding proteins [4]. This interaction was further supported by thermostability measurements conducted using differential scanning fluorimetry (SUPR‐DSF), which showed a clear increase in melting temperature (T_m_) upon FMN binding (63.6°C to 66.5°C), indicating a stabilizing effect of FMN on PhdC (Figure S4, Table S2). Together, these data confirm that a PhdC:FMN complex is readily formed in solution and suggest a similar complex is likely formed with prFMN species.

### 3.2 Characterization of PhdC‐Mediated prFMN Oxidative Maturation

**FIGURE 3:**
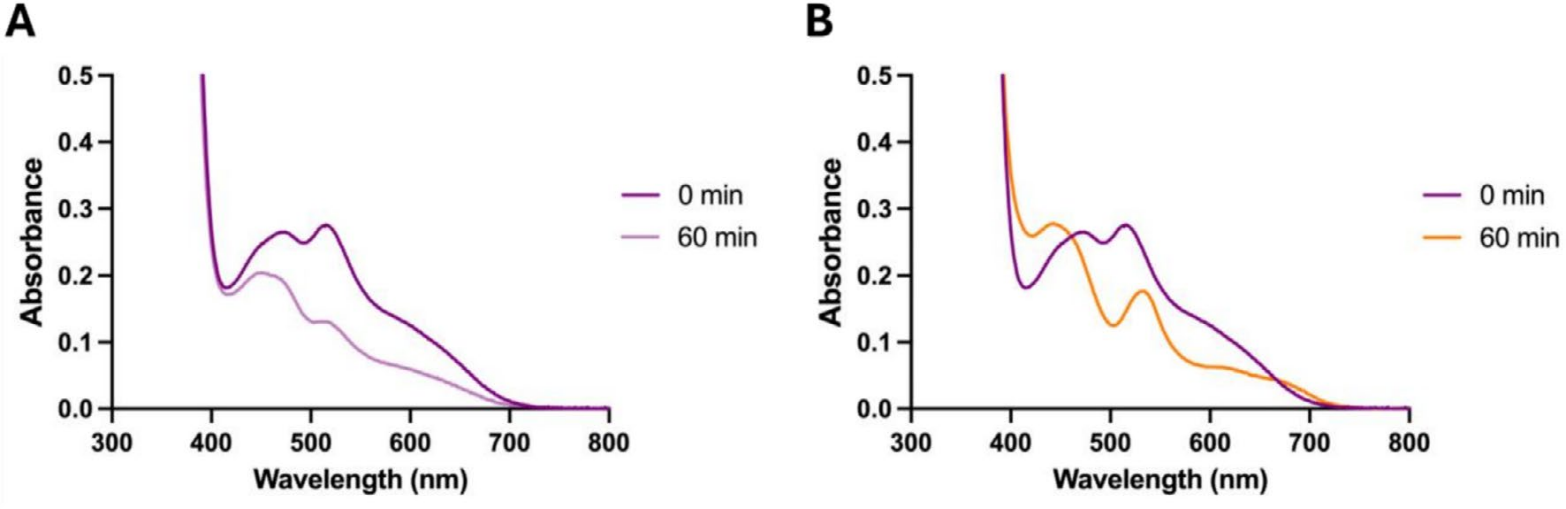
PhdC mediated conversion of prFMN^radical^ under aerobic conditions. (A) UV–vis spectra of ~100 μM free prFMN^radical^ at 0 and 60 min following oxygen exposure. (B) UV–vis spectra of ~100 μM free prFMN^radical^ incubated with ~50 μM PhdC at 0 and 60 min following oxygen exposure.

**FIGURE 4:**
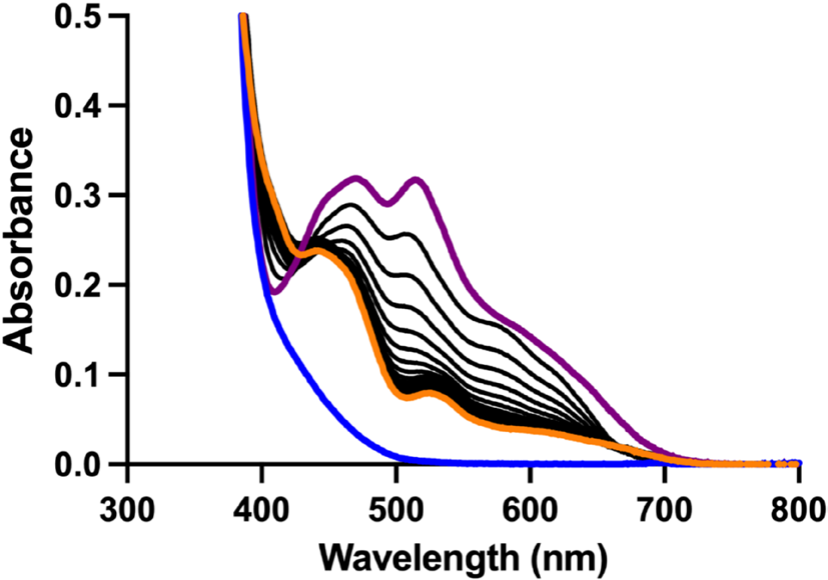
PhdC‐mediated prFMN^radical^ oxidation under semi‐anaerobic conditions. Overlay of UV–visible spectra of ~100 μM reduced prFMNH_2_ (in blue), rapid formation of the prFMN^radical^ upon aerobic exposure (in purple), and gradual conversion of the radical to the oxidized iminium species (in orange) following incubation with ~50 μM PhdC (in black, individual spectra taken at 2 min intervals revealing the gradual transition between the purple and orange spectral species).

Reduced prFMNH_2_ was generated under anaerobic conditions as described in the methods section. Upon oxygen exposure, the solution turned purple, concordant with the formation of the prFMN^radical^ species [6]. Spectral analysis revealed two peaks at 490 and 515 nm, previously attributed to distinct protonation states of the free prFMN^radical^ species (Figure 3A) [23]. Gradual bleaching of these features occurs over a 60‐min period, indicating instability of the radical species under aerobic conditions. In contrast, when incubated with PhdC, the prFMN^radical^ spectrum shifted toward peaks at 450 and 535 nm over the same timeframe, the former of which is consistent with the formation of the catalytically active prFMN^iminium^ species in solution (Figure 3B). Concomitantly, the solution changed color from bright purple to pale yellow, further supporting oxidative conversion of the radical intermediate to the active iminium form. In contrast, when kept under anaerobic conditions, UV–visible spectroscopy of the prFMNH_2_ solution yielded features consistent with previously reported data (Figure 4, blue trace) [9]. Following oxygen exposure, the resulting purple prFMN^radical^ solution was returned to the anaerobic glove box (oxygen concentration at < 10 ppm) (Figure 4, purple trace) [23]. Purified PhdC was rendered anaerobic and added to the anaerobic prFMN^radical^ solution at a final concentration of 50 μM. Over a 2‐h time course, the spectrum shifted toward ~450 nm, with a less pronounced peak at 535 nm (Figure 4, yellow trace), consistent with the formation of the catalytically active prFMN^iminium^ species in solution and concomitant color transition from purple to yellow. These results demonstrate that PhdC facilitates prFMN^radical^ oxidative maturation even under semi‐anaerobic conditions (oxygen concentration at < 10 ppm).

**FIGURE 5:**
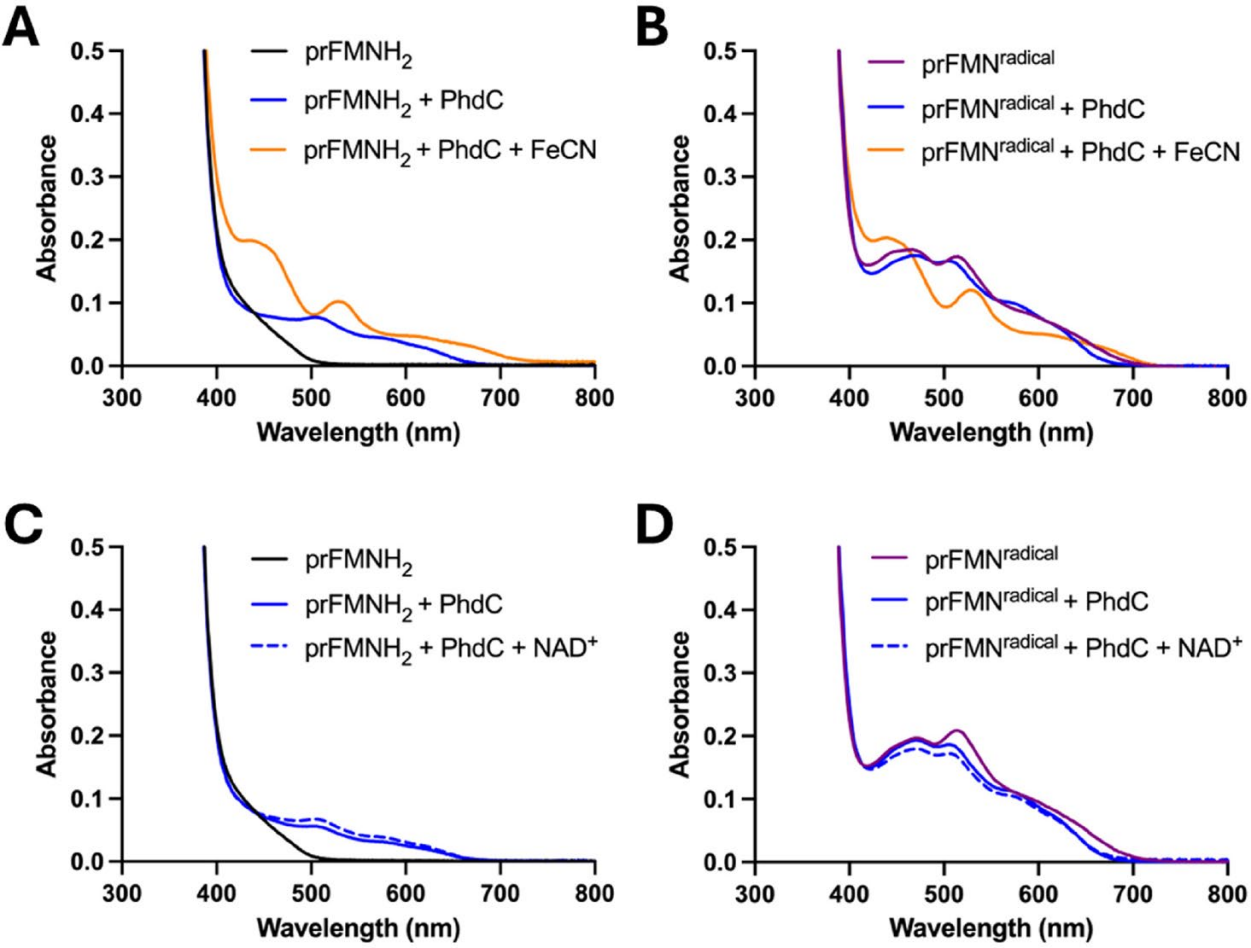
PhdC‐catalyzed prFMN^radical^ maturation with alternative oxidants. Reactions carried out in the presence of glucose oxidase and catalase +10 mM glucose under a nitrogen atmosphere. (A) prFMNH_2_ in the presence of PhdC and 2 M equivalents of potassium ferricyanide (FeCN). (B) prFMN^radical^ in the presence of PhdC and 2 M equivalents of potassium ferricyanide (C) prFMNH_2_ in the presence of PhdC and 1 mM of NAD+ (D) prFMN^radical^ in the presence of PhdC and 1 mM of NAD+.

When experiments were carried out in the presence of glucose oxidase and catalase with buffers supplemented with glucose to ensure complete removal of all traces of oxygen, no prFMN oxidation was observed in the presence of PhdC (Figure 5). When alternative oxidants were added, activity was observed in the presence of potassium ferricyanide (K_3_[Fe(CN)_6_]), but not NAD^+^. Potassium ferricyanide promotes rapid formation of prFMN^radical^ in the absence of PhdC (Figure S5), but does not support conversion to the prFMN^iminium^ species. Thus, PhdC catalyzes prFMN^radical^ oxidation in the presence of oxygen or (K_3_[Fe(CN)_6_]).

### 3.3 Kinetic Characterization of PhdC‐Catalyzed Oxidation of prFMN^radical^

**FIGURE 6:**
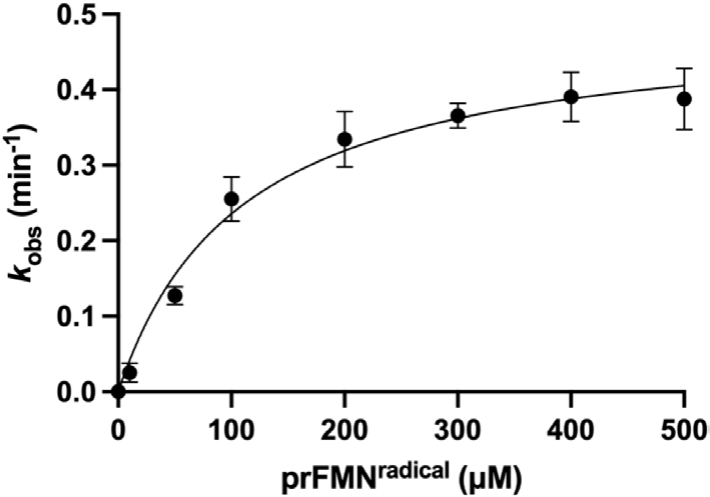
Kinetics of PhdC‐catalyzed prFMN^radical^ maturation. PhdC activity toward prFMN^radical^. Reaction monitored by consumption of free prFMN^radical^ substrate at 515 nm by 50 μM PhdC under semi‐anaerobic conditions (< 10 ppm O_2_).

PhdC activity toward prFMN^radical^ was examined under semi‐anaerobic conditions to avoid issues with prFMN^radical^ bleaching observed under aerobic conditions by monitoring the decrease in absorbance at 515 nm, corresponding to consumption of the radical species [23]. Rates were determined at varying prFMN^radical^ concentrations and subsequently fitted to a first‐order dependence to describe the overall kinetic behavior. The observed rate constant was determined to be *k*_obs_ = 0.49 ± 0.03 min^−1^ (Figure 6). The modest rate observed under these conditions likely reflects limited oxygen availability.

### 3.4 PhdC Co‐Expression Activates prFMN‐Dependent Canonical UbiD

**FIGURE 7:**
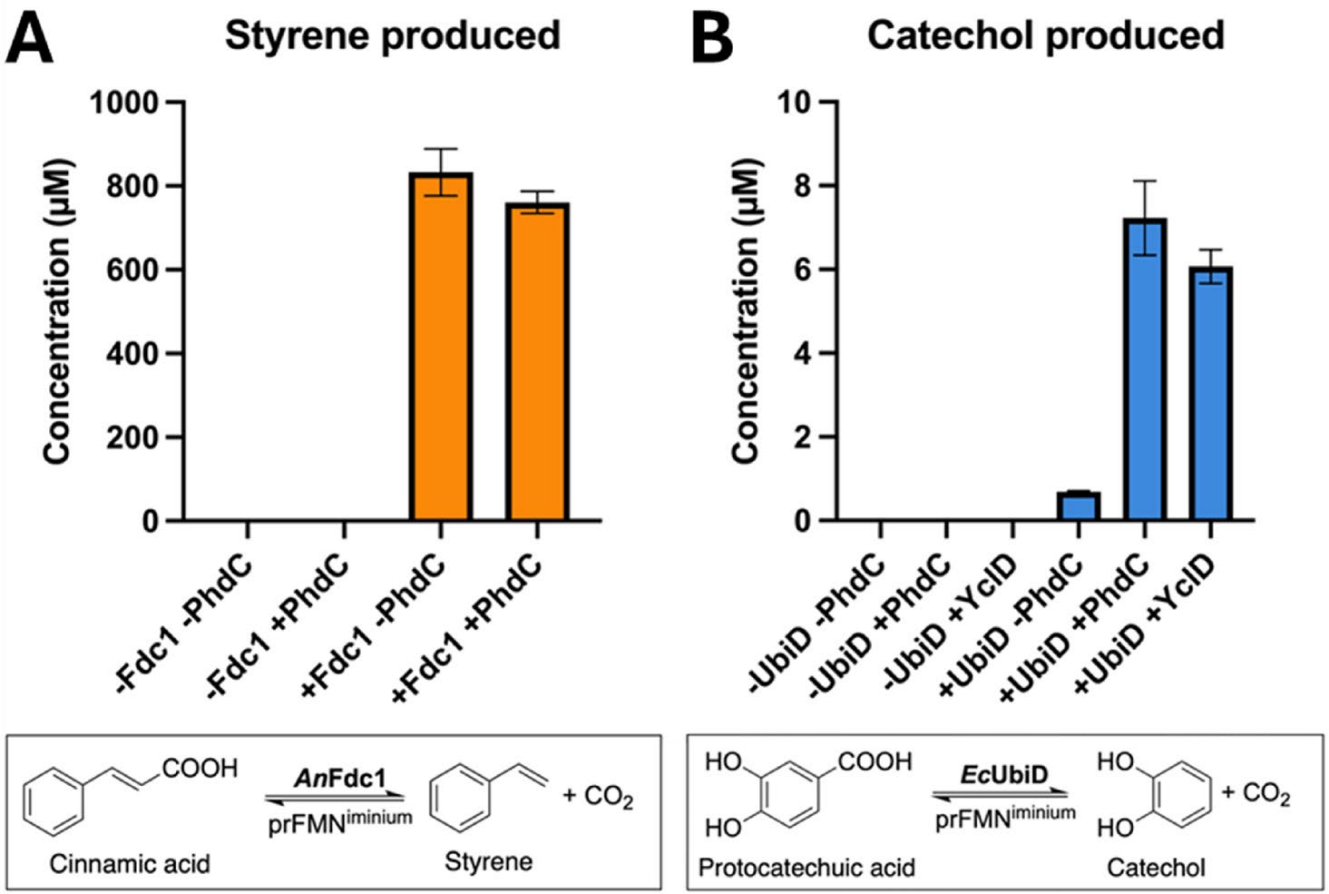
Effect of PhdC co‐expression on UbiD‐expressing whole cell decarboxylase activity. (A) Styrene production levels indicating cinnamic acid decarboxylase activity *of E. coli* BL21 (DE3) whole cells expressing *An*Fdc1 with or without PhdC. (B) Catechol production levels indicating protocatechuic acid decarboxylase activity *of E. coli* BL21 (DE3) whole cells overexpressing EcUbiD with or without PhdC or the distant homologue YclD. Samples were prepared in triplicate and analyzed using HPLC.

Co‐expression of PhdC with the model UbiD enzyme *An*Fdc1 and UbiX does not significantly enhance the *An*Fdc1‐mediated decarboxylation of cinnamic acid (Figure 7A). Similar yields of the styrene product were obtained for cells co‐expressing PhdC with *An*Fdc1 and UbiX and those expressing *An*Fdc1 and UbiX alone. In contrast, co‐expression of PhdC with the canonical *Ec*UbiD and UbiX resulted in a 10.4‐fold increase in the production of catechol from protocatechuic acid, compared to cells lacking PhdC (Figure 7B). These results demonstrate that PhdC can restore or enhance catalytic activity in UbiD enzymes that require activation of the prFMN cofactor to the catalytically active iminium species, confirming its functional role in oxidative maturation under physiological conditions. In addition, we tested the effect of an alternative PhdC homologue, YclD from *Priestia megaterium* (31.5% similarity) that is associated with the VdcCD enzymes [24, 25], and found YclD co‐expression had a similar effect on *Ec*UbiD activity, prompting an 8.8‐fold increase in catechol production relative to cells expressing *Ec*UbiD and UbiX alone.

### 3.5 PhdC Crystal Structure Determination

**FIGURE 8:**
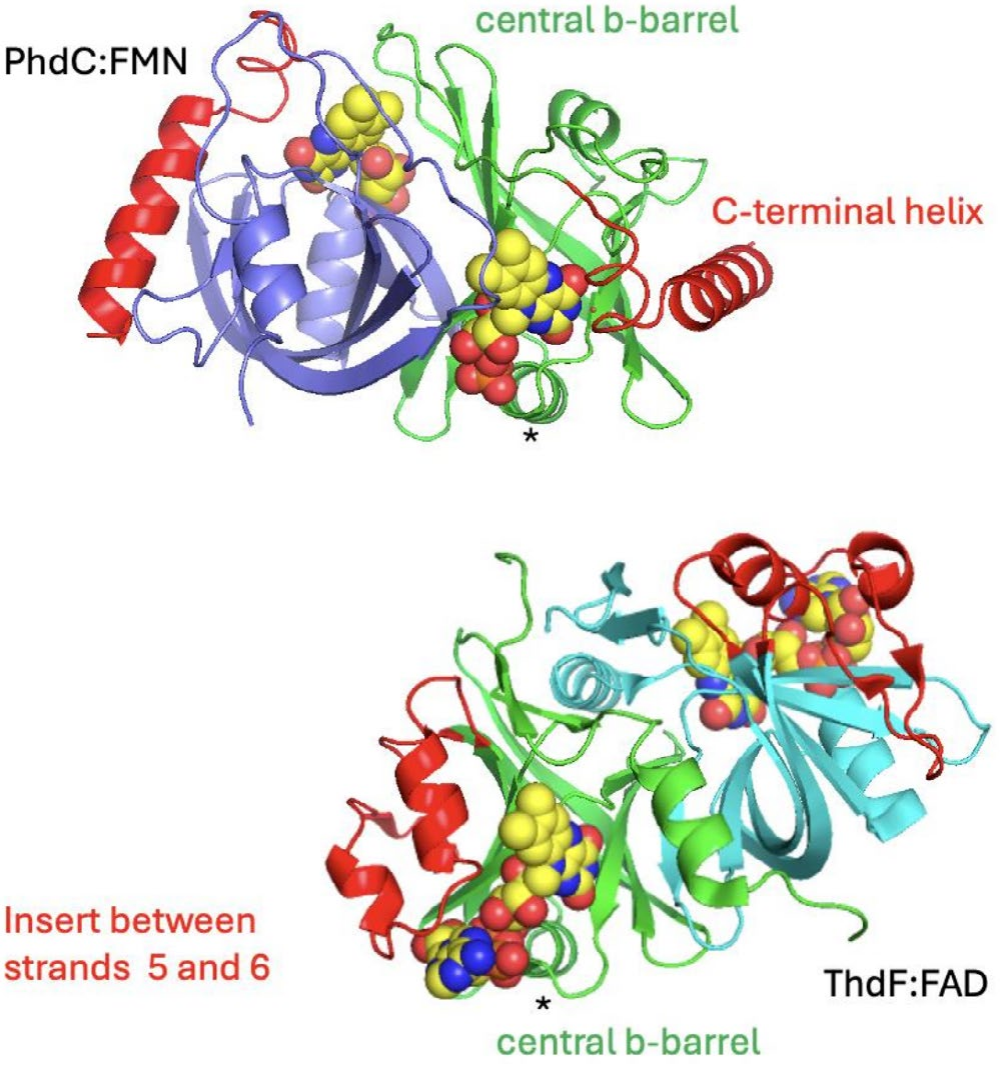
Structure of PhdC. Cartoon representation of PhdC in complex with FMN shown alongside the structurally related ThdF in complex with FAD (PDB: 9FD4) [27]. Both monomers consist of a central beta‐barrel (in green) binding the flavin substrate with an alpha‐helix connecting strands 4 and 5 directly interacting with the FMN/FAD phosphate(s) as indicated by a *. The dimer interface formed in each protein is however drastically different, with the PhdC C‐terminal helix occupying the interface formed between individual monomer beta‐barrels in ThdF. In the latter, a large insertion between strands 5 and 6 occupies the interface formed between individual monomer beta barrels in PhdC. As a direct consequence, the flavin isoalloxazine environment is highly distinct with the PhdC active site occurring at the dimer interface.

The PhdC structure reveals it belongs to the class I HpaC‐like family of short‐chain dimeric flavin reductases, despite having low sequence homology and lacking the key SxxPP and GDH motifs associated this family [26]. One of the closest structural homologues in the PDB is the NADH‐dependent flavin reductase ThdF (PDB code 9FD5) [27] with a Z score of 10.9 and r.m.s.d. of 2.5 Å over 103 Calphas (sequence homology 14%, Figure 8). While the flavin binding mode is similar, located at the surface of the protein with the phosphate bound at the N‐terminus of the helix connecting beta strands 4 and 5 of the central beta barrel, the dimerization mode is distinct. In case of PhdC, the extensive dimer interface (2888 A^2^ buried surface area) is formed by juxtaposition of the individual monomer beta barrels strands 1, 2, 5 and 6. In contrast, the HpaC‐like ThdF dimer interface is formed by interaction between beta strands 3, 4, 6 and 7 in addition to the N‐ and C‐terminal regions. In ThdF, an extensive insert between beta strands 5 and 6 (Figure 8, in red) replaces the PhdC‐dimer interface, while in PhdC the C‐terminal helix is positioned at the ThdF‐dimer interface adjacent to strands 4, 6 and 7.

**FIGURE 9:**
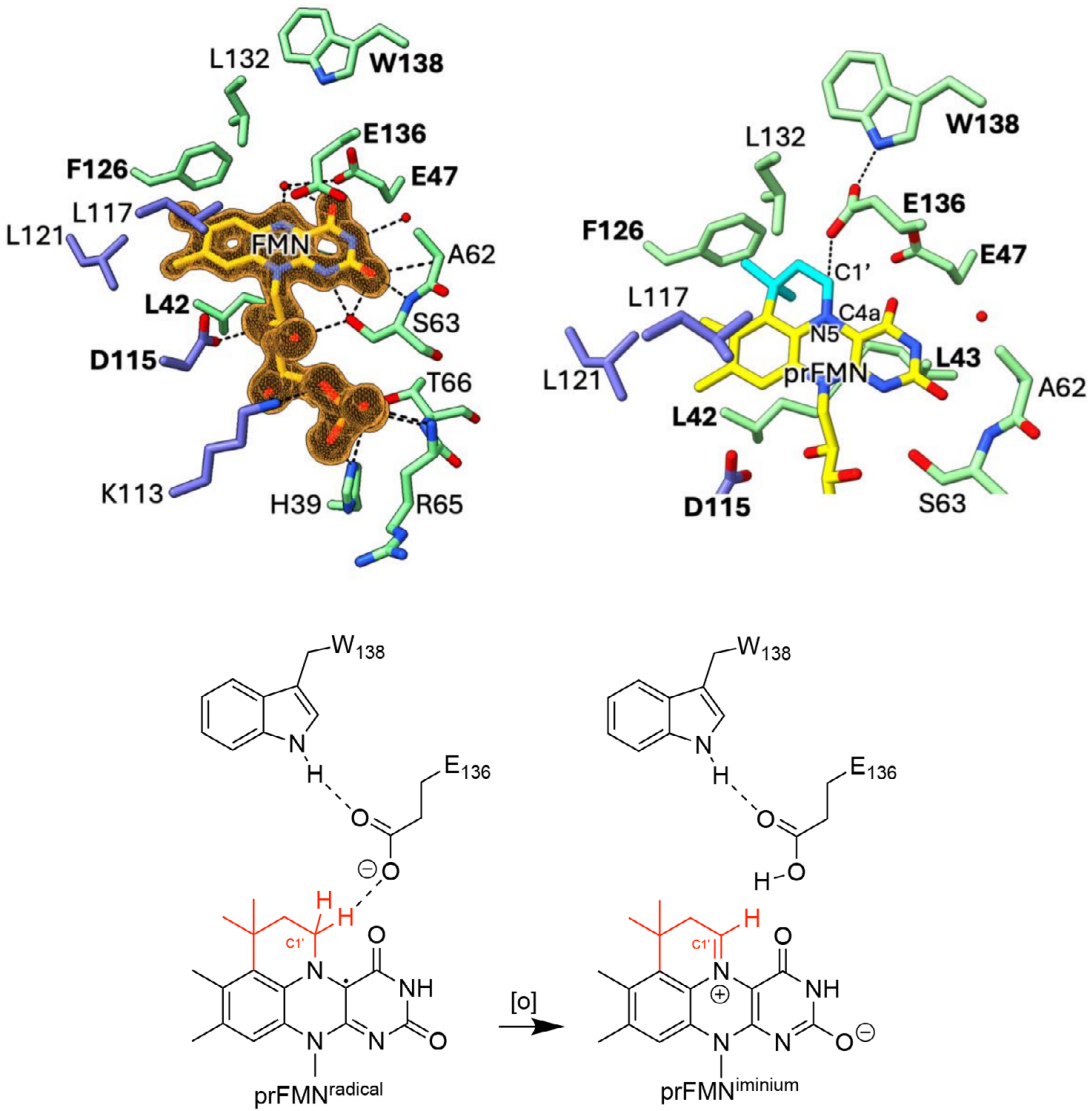
PhdC FMN‐binding site. (A) Key amino acids interacting with bound the FMN as shown in atom colored sticks, monomer A in green monomer B in blue. Polar interactions are shown by gray dotted lines. Highly conserved residues are labeled in bold (alignment see Figures S6 and S7). Omit map electron density for the bound flavin is shown in orange contoured at 3 sigma. (B) Model of the PhdC‐prFMN interaction with the prenyl group shown in cyan and Glu136 modeled in an alternative conformation. (C) Proposed mechanism for prFMN^radical^ oxidation in PhdC.

While comparison between monomer A and B from the *apo*‐structure reveals both are similar in conformation (rmsd 0.33 Å) comparison with the FMN‐bound structure reveals minor changes at the isoalloxazine binding site, consistent with ligand binding induced ordering of loops. The loop region preceding the C‐terminal helix centered around Glu136 undergoes the largest shift (~2.5 Å) in position. The isoalloxazine ring forms hydrophobic interactions with L41, L42, L121 (monomer B), L117 (monomer B), L132 and direct polar interactions with Glu47, the main chain of Al62, Ser 63, in addition to the Glu136 carboxylate side chain forming a pi‐stacking interaction (Figure 9A). Sequence alignments reveal the majority of these residues are conserved, with Glu136 strictly conserved as part of a C‐terminal ExW motif (Figures S6 and S7). Modeling of the prFMN^radical^ complex can easily be achieved by extending the FMN with the prenyl‐ring for which there is a hydrophobic binding pocket formed by F126 and L132 (Figure 9B). This reveals the C1′ can be brought into direct hydrogen bonding contact with the Glu136 residue when the latter is modeled in the different conformer. In this case, a direct hydrogen bonding interaction is formed between Glu136 and Trp138. This suggests PhdC catalyzes prFMN^radical^ oxidation by proton abstraction from C1′ via the Glu136 residue (Figure 9C). The latter is in close proximity of Glu47, an interaction that might serve to optimize the Glu136 pK_a_.

## 4 Discussion

In view of the difficulty associated with isolating pure forms of prFMN, and their instability in solution, PhdC binding to FMN was assessed as a model for prFMNH_2_ binding. The corresponding dissociation constant (K_d_ = 17.75 ± 0.45 μM) is comparable to that observed for *Pa*UbiX [6], but ~10‐fold weaker than the Mn^2+^‐dependent *Ec*UbiD–FMN interaction [4]. This is consistent with the need for prFMN species produced by UbiX and subsequently matured in PhdC to ultimately be bound by the UbiD enzyme.

The PhdC mediated conversion of prFMN^radical^ was confirmed spectroscopically under both aerobic and semi‐anaerobic conditions. Crucially, no prFMN^radical^ conversion is observed in absence of PhdC. Under semi‐anaerobic conditions (oxygen concentration in atmosphere estimated at < 10 ppm), prFMN^radical^ conversion by PhdC yielded *k*_obs_ = 0.49 ± 0.03 min^−1^ in the presence of excess prFMN^radical^. This aligns with the slow aerobic reaction previously described by DiRocco and colleagues [5], reinforcing the role of prFMN^radical^ as the substrate of the PhdC catalyzed oxidative maturation reaction. Under strict anaerobic conditions, potassium ferricyanide (but not NAD^+^) can serve as alternative oxidant to generate prFMN^iminiun^ from prFMNH_2_ or prFMN^radical^ in presence of PhdC(Figure 5). While prFMNH_2_ is rapidly oxidized by potassium ferricyanide or oxygen to the prFMN^radical^ state, efficient further oxidation to the prFMN^iminium^ species requires the presence of PhdC. The exact nature of the physiological oxidant for strict anaerobes remains unclear.

We tested the hypothesis proposed by DiRocco et al. (2024) [5] that PhdC can enhance the decarboxylative activity of heterologously (over)expressed UbiD enzymes, by assessing the effect on whole‐cell mediated decarboxylation of cinnamic acid by *An*Fdc1 or protocatechuic acid by *Ec*UbiD respectively. The model system *An*Fdc1 is readily isolated or reconstituted with the active prFMN^iminium^ species likely due to efficient autocatalysis of the oxidative maturation process. In contrast to the canonical *Ec*UbiD which cannot readily be isolated or reconstituted in active form [4, 10] As expected, no significant increase in cinnamic acid decarboxylation activity was observed for cells expressing *An*Fdc1 with PhdC. In contrast, co‐expression of PhdC with *Ec*UbiD resulted in a 10.4‐fold increase in decarboxylation of PCA to catechol. These results further demonstrate that PhdC can enhance the catalytic activity of prFMN‐dependent UbiD enzymes with inefficient or missing autocatalytic oxidative maturation, and confirms its functional role as a prFMN oxidative maturase in vivo. The findings are consistent with those of DiRocco and colleagues (2024) [5], who reported a similar enhancement when co‐expressing the UbiD homologue *Pt*HmfF with *Mf*PhdB (a UbiX homologue) and PhdC.

In light of this, we selected *Pm*YclD, a small putative UbiDX accessory protein for further analysis. YclD shares approximately 30% sequence similarity with PhdC. In vivo assays reveal that co‐expression of *Ec*UbiD with *Pm*YclD resulted in an 8.8‐fold increase in catechol formation relative to *Ec*UbiD alone, demonstrating that *Pm*YclD enhances activity of the canonical *Ec*UbiD in a manner analogous to PhdC. According to a BLAST search using the clustered non‐redundant database, there are presently 86 PhdC‐like sequences of similar length. All sequences belong to the phylum *Actinomycetota*, with the most frequent genera consisting of *Nocardia* and *Actinophytocola*. Percent identity of these sequences with respect to PhdC varies broadly from ~35%–90%. Despite this, several residues with more than 90% conservation were identified in the active site (Figure S6). Some, such as Glu47, Glu136 and Trp138 (PhdC numbering) may be directly involved in catalysis while others such as Phe126 and the consistently hydrophobic residue at position 132 likely accommodate the prenyl ring of prFMN. *Pm*YclD was included in sequence analysis despite its absence from the BLAST search results due to its PhdC‐like UbiD‐activating nature and has a low percentage identity with PhdC (17%). Despite this, Glu47 and Glu136 are conserved, while Trp138 is not.

The PhdC crystal structure in complex with flavin allows modeling of the PhdC‐prFMN complex, revealing conserved residues located near the C1′‐N5‐C4a locus that is the subject of the oxidative maturation process. Given conversion of the prFMN^radical^ species is associated with C1′ deprotonation, catalysis of the oxidative maturation process likely occurs through positioning of an acid–base catalyst in close proximity. In this case, both the conserved Glu47 and 136 are positioned near the C1′, with Glu136 readily positioned within hydrogen bonding distance. Unraveling the exact mechanism of PhdC will require further studies on a range of wild type and variant enzymes. Solution data combined with structural insights into the *M. fortuitum* enzyme suggest acid–base mediated C1′ deprotonation combined with electron transfer from the C1′‐N5‐C4a locus is central to the oxidative process (Figure 9C). To what extent the process is optimized for electron transfer from the prFMN^radical^ species as opposed to two‐electron transfer from the prFMNH_2_ species remains unclear. The rapid oxidation of prFMNH_2_ to the radical form in presence of oxygen makes studying the effect of proteins on the latter process challenging. This work further confirms PhdC co‐expression can result in higher activity levels during heterologous expression UbiD enzymes, and adds PhdC to the expanding repertoire of prFMN‐binding proteins associated with the widespread UbiDX system.

## Author Contributions

**Dominic R. Whittall:** writing – original draft, writing – review and editing, investigation, formal analysis. **Henry G. Box:** writing – review and editing, investigation, formal analysis, writing – original draft. **Karl A. P. Payne:** writing – review and editing, investigation. **Stephen A. Marshall:** investigation. **David Leys:** writing – review and editing, project administration, supervision, funding acquisition.

## Funding

This work was supported by the Biotechnology and Biological Sciences Research Council (BBSRC) grant BB/W016745/1 and by BBSRC/EPSRC Prosperity Partnership grant Sustainable Commodity Chemicals through Enzyme Engineering & Design (SuCCEED) BB/Y003276/1 (both to D.L.).

## Conflicts of Interest

The authors declare no conflicts of interest.

## Supporting information


**Data S1:** Supporting Information.

## Data Availability

The atomic coordinate and structure factors (pdb codes: 9SRG and 9SRJ) have been deposited to the Protein Data Bank (http://www.pdb.org).
