# Supplementary material for: Structure and Mechanism of PhdC, a Prenylated‐Flavin Maturase
Source: Proteins. 2025 Dec 9;94(5):1019–29. doi: 10.1002/prot.70096 (PMC13040415; doi:10.1002/prot.70096)
Supplement: Supplementary file 1 — Data S1: Supporting Information. [file PROT-94-1019-s001.docx]

Supplementary information: Structure and Mechanism PhdC, a Prenylated Flavin Maturase

**Dominic R. Whittall^1†^, Henry G. Box^1†^, Karl A.P. Payne^1^, Stephen A. Marshall^1,2^, David Leys^1^***

^1^Manchester Institute of Biotechnology, University of Manchester, Princess Street 131 Manchester, M1 7DN, UK.

^2^present address: Department of Chemistry, Chemistry Research Laboratory, University of Oxford, Mansfield Road, Oxford OX1 3TA, UK & Ineos Oxford Institute for Antimicrobial Research, University of Oxford, South Parks Road, Oxford OX1 3RE, UK

**Figure S1. SDS-PAGE of PhdC following purification by Ni-NTA and PD-10 desalting.**

**
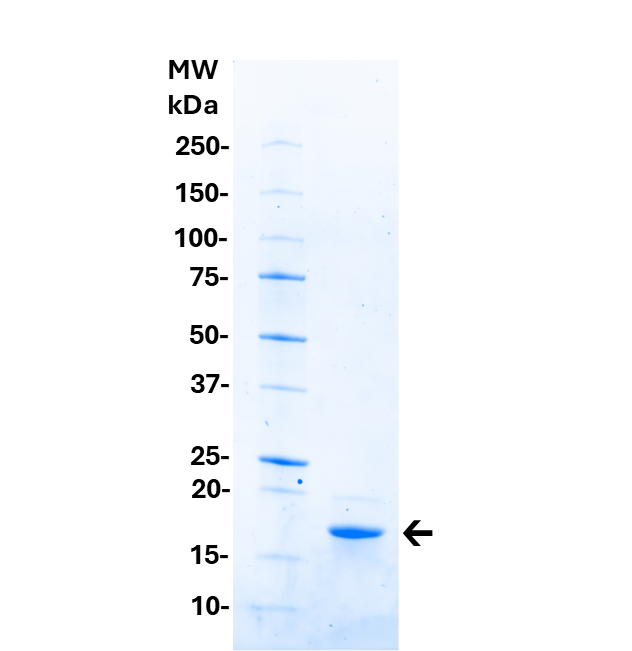
**

**Figure S2. Calibration curve used for the determination of free prFMN^radical^ extinction coefficient at 515 nm.**

**
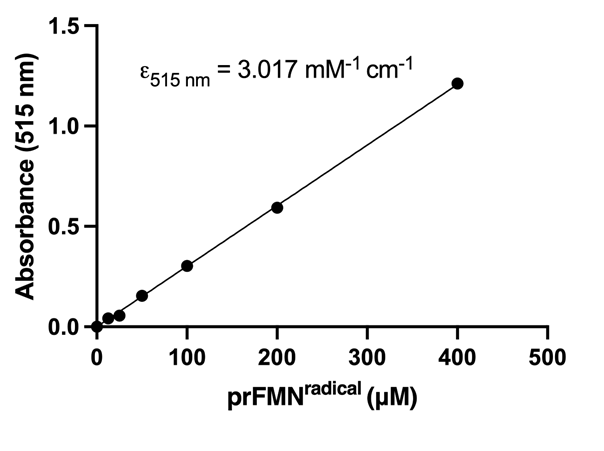
Figure S3: ITC binding saturation curves for PhdC with FMN.**

**
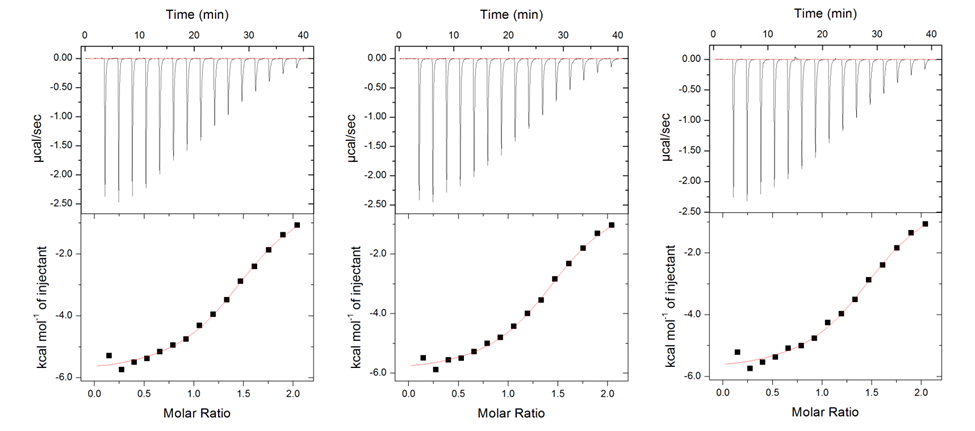
**

**Table S1: Calculated association and dissociation constants of ITC experiments for PhdC with FMN.**

| **Replicate** | **K_a_ (M^-1^)** | **K_d_ (µM)** |
| --- | --- | --- |
| **1** | 57400 | 17.42 |
| **2** | 56930 | 17.57 |
| **3** | 54760 | 18.26 |
| **mean** | 56363 | 17.75 |
| **std dev** | 1408 | 0.45 |
| **std error** | 813 | 0.26 |

**Figure S4. Thermostability of PhdC in absence (green) and presence (orange) of FMN.** A) Barycentric mean of the shift in tryptophan fluorescence due to protein unfolding across the temperature gradient. Dotted and coloured lines indicate region used to model statistics. B) Fraction of protein unfolded across a 20 – 90 °C gradient. Sigmoidal curves are representative replicates of the mean values (dotted lines).

**
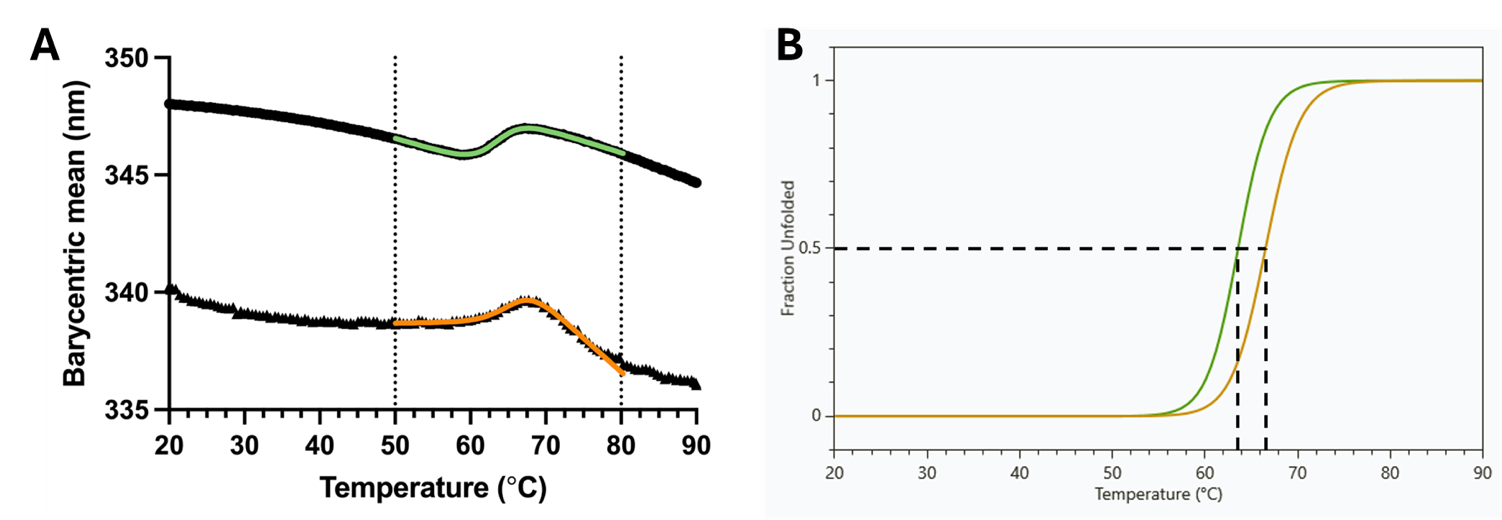
Figure S5:** Potassium ferricyanide mediated conversion of prFMN species under strict anaerobic conditions. **A.** UV-vis spectra of ~100 μM free prFMNH_2_ following addition of K_3_[Fe(CN)_6_]. **B.** UV-vis spectra of ~100 μM prFMN^radical^ incubated with following addition of K_3_[Fe(CN)_6_].

**
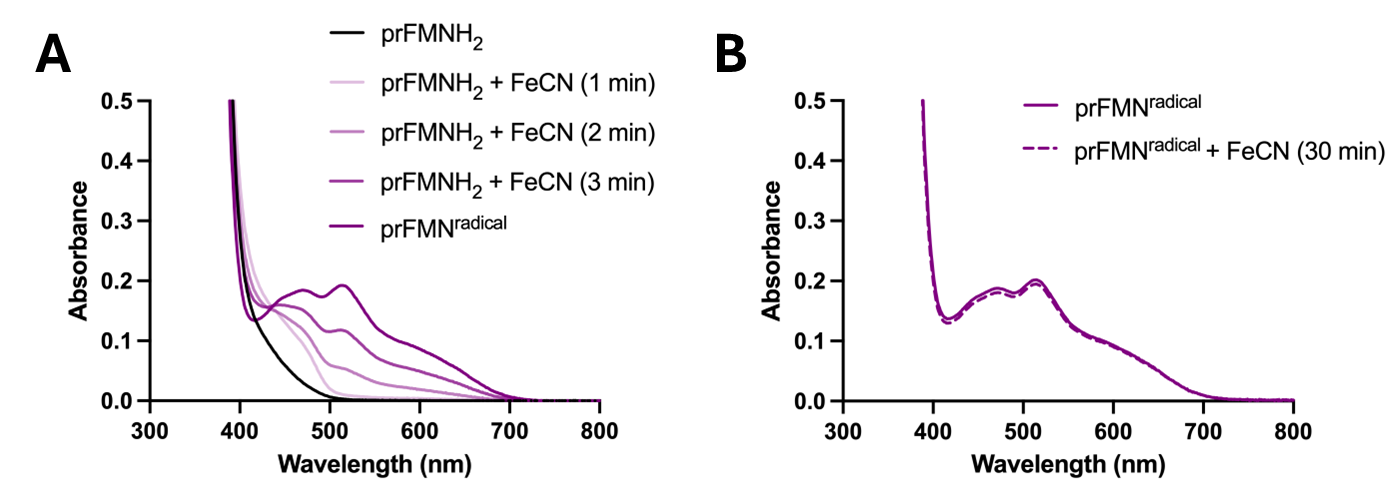
**

**Figure S6. Multiple Sequence Alignment (MSA) of PhdC and homologue sequences**. Sequence numbers are annotated according to *M. fortuitum* PhdC. MSA generated using sequences retrieved via NCBI BLAST from the ClusteredNR database. MSA visualised and consensus sequence calculated using Jalview.

**
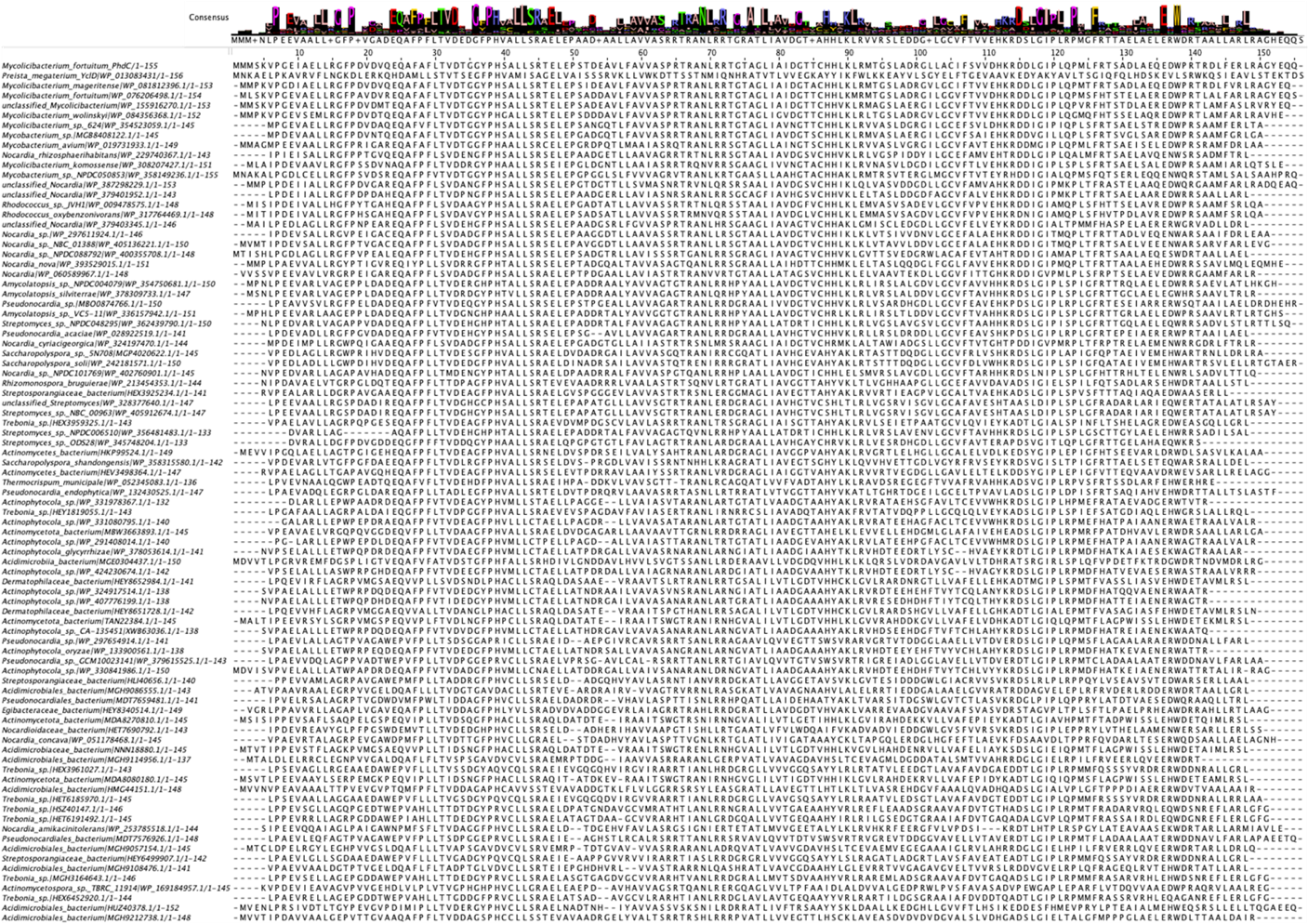
**

**Figure S7.** **Phylogenetic tree of *Mf*PhdC (red), *Pm*YclD (green), and homologous sequences.** Tree constructed using multiple sequence alignment presented in **Figure S6**. Despite being among the most distant sequences on the tree, both PhdC and YclD boost whole-cell *Ec*UbiD activity.

**
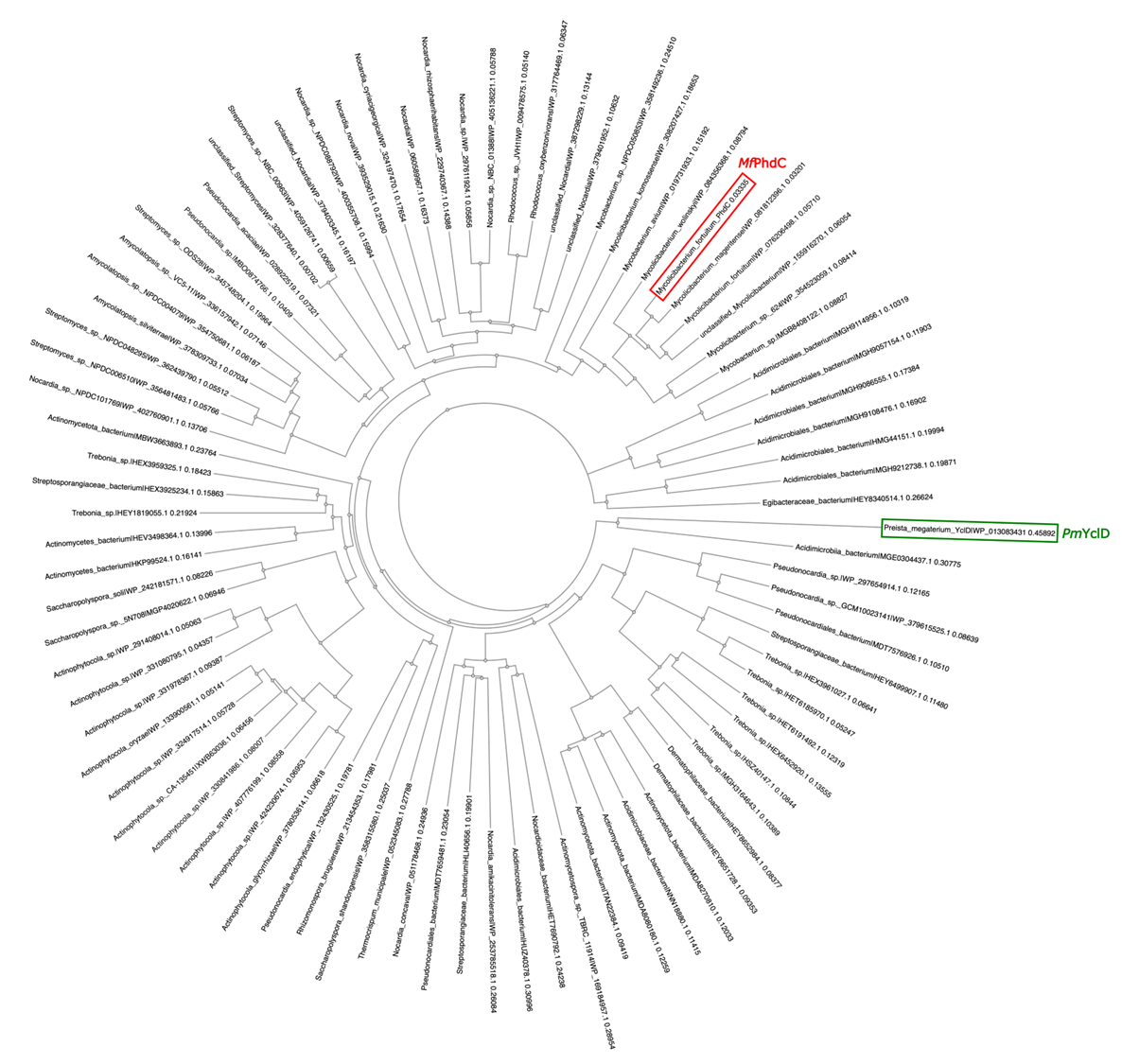
**

**Table S2: Thermostability data for PhdC in the *apo*- and FMN-bound states determined using differential scanning fluorimetry.**

| **Sample** | **T_on_ (°C)** | **T_m_ (°C)** | **dH (kJ/mol)** |
| --- | --- | --- | --- |
| ***Apo*-PhdC** | 55.93 ± 0.30 | 55.93 ± 0.30 | 55.93 ± 0.30 |
| **FMN-PhdC** | 57.38 ± 0.82 | 57.38 ±0.82 | 57.38 ± 0.82 |
